# Supplementary material for: CRISPR screens in 3D tumourspheres identified miR-4787-3p as a transcriptional start site miRNA essential for breast tumour-initiating cell growth
Source: Commun Biol. 2024 Jul 13;7:859. doi: 10.1038/s42003-024-06555-1 (PMC11246431; doi:10.1038/s42003-024-06555-1)
Supplement: Supplementary file 2 — Supplementary Information [file 42003_2024_6555_MOESM2_ESM.pdf]

Figure S1

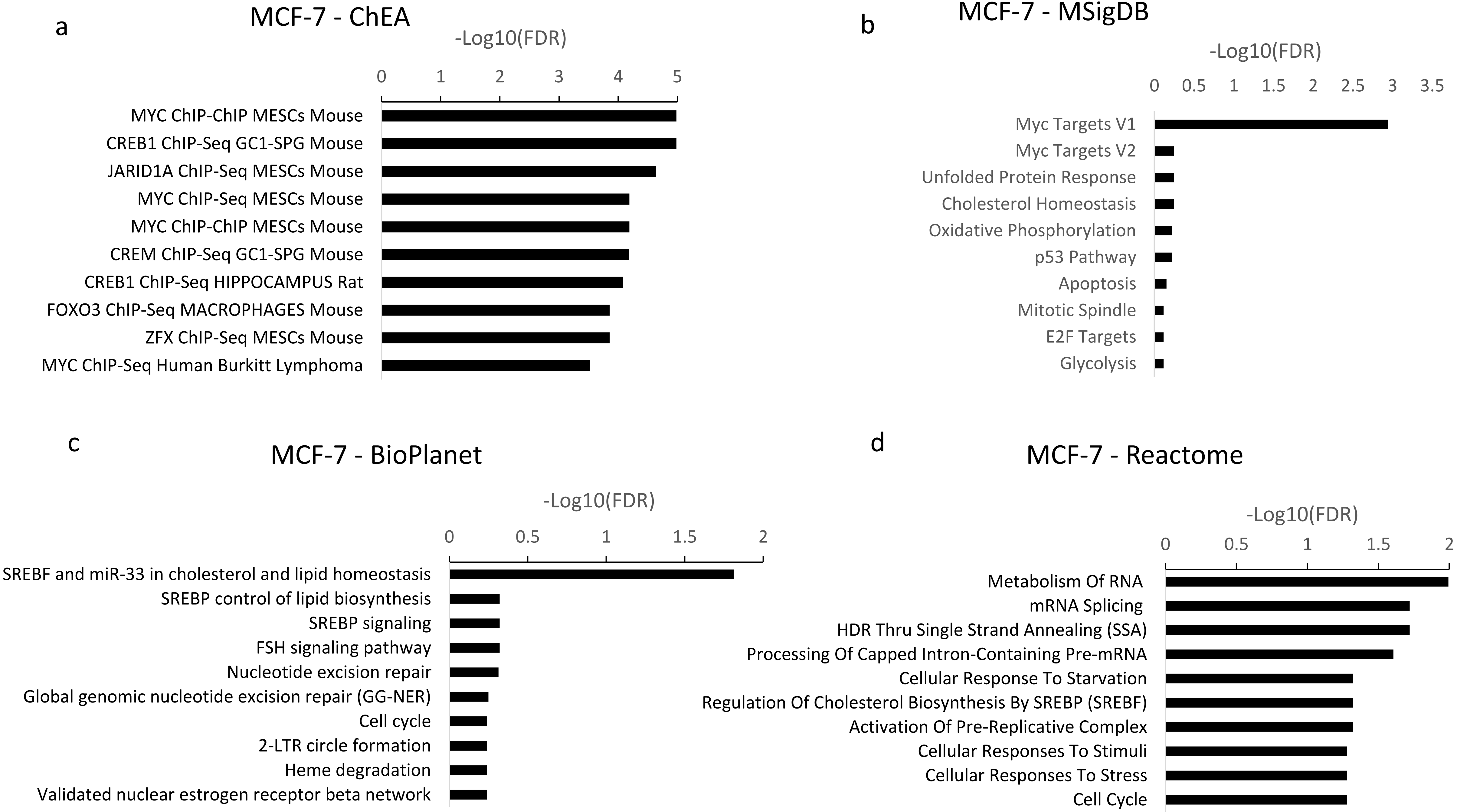
a
MCF-7 - ChEA
-Log10(FDR)
0
1
2
3
4
5
MYC ChIP-ChIP MESCs Mouse
CREB1 ChIP-Seq GC1-SPG Mouse
JARID1A ChIP-Seq MESCs Mouse
MYC ChIP-Seq MESCs Mouse
MYC ChIP-ChIP MESCs Mouse
CREM ChIP-Seq GC1-SPG Mouse
CREB1 ChIP-Seq HIPPOCAMPUS Rat
FOXO3 ChIP-Seq MACROPHAGES Mouse
ZFX ChIP-Seq MESCs Mouse
MYC ChIP-Seq Human Burkitt Lymphoma
b
MCF-7 - MSigDB
-Log10(FDR)
0
0.5
1
1.5
2
2.5
3
3.5
Myc Targets V1
Myc Targets V2
Unfolded Protein Response
Cholesterol Homeostasis
Oxidative Phosphorylation
p53 Pathway
Apoptosis
Mitotic Spindle
E2F Targets
Glycolysis
c
MCF-7 - BioPlanet
-Log10(FDR)
0
0.5
1
1.5
2
SREBF and miR-33 in cholesterol and lipid homeostasis
SREBP control of lipid biosynthesis
SREBP signaling
FSH signaling pathway
Nucleotide excision repair
Global genomic nucleotide excision repair (GG-NER)
Cell cycle
2-LTR circle formation
Heme degradation
Validated nuclear estrogen receptor beta network
d
MCF-7 - Reactome
-Log10(FDR)
0
0.5
1
1.5
2
Metabolism Of RNA
mRNA Splicing
HDR Thru Single Strand Annealing (SSA)
Processing Of Capped Intron-Containing Pre-mRNA
Cellular Response To Starvation
Regulation Of Cholesterol Biosynthesis By SREBP (SREBF)
Activation Of Pre-Replicative Complex
Cellular Responses To Stimuli
Cellular Responses To Stress
Cell Cycle

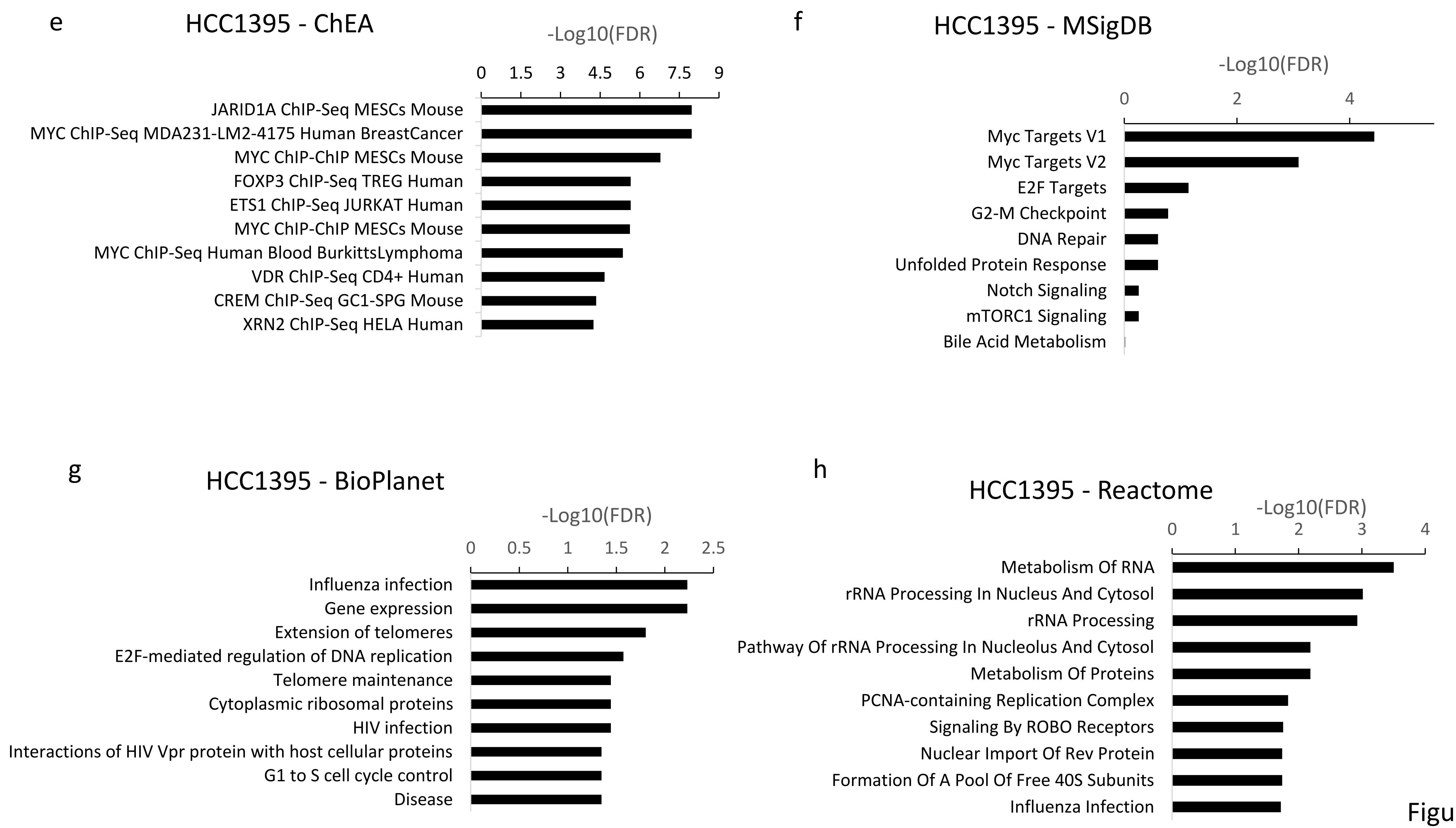

e
HCC1395 - ChEA
-Log10(FDR)
0 1.5 3 4.5 6 7.5 9
JARID1A ChIP-Seq MESCs Mouse
MYC ChIP-Seq MDA231-LM2-4175 Human BreastCancer
MYC ChIP-ChIP MESCs Mouse
FOXP3 ChIP-Seq TREG Human
ETS1 ChIP-Seq JURKAT Human
MYC ChIP-ChIP MESCs Mouse
MYC ChIP-Seq Human Blood BurkittsLymphoma
VDR ChIP-Seq CD4+ Human
CREM ChIP-Seq GC1-SPG Mouse
XRN2 ChIP-Seq HELA Human
f
HCC1395 - MSigDB
-Log10(FDR)
0 2 4
Myc Targets V1
Myc Targets V2
E2F Targets
G2-M Checkpoint
DNA Repair
Unfolded Protein Response
Notch Signaling
mTORC1 Signaling
Bile Acid Metabolism
g
HCC1395 - BioPlanet
-Log10(FDR)
0 0.5 1 1.5 2 2.5
Influenza infection
Gene expression
Extension of telomeres
E2F-mediated regulation of DNA replication
Telomere maintenance
Cytoplasmic ribosomal proteins
HIV infection
Interactions of HIV Vpr protein with host cellular proteins
G1 to S cell cycle control
Disease
h
HCC1395 - Reactome
-Log10(FDR)
0 1 2 3 4
Metabolism Of RNA
rRNA Processing In Nucleus And Cytosol
rRNA Processing
Pathway Of rRNA Processing In Nucleolus And Cytosol
Metabolism Of Proteins
PCNA-containing Replication Complex
Signaling By ROBO Receptors
Nuclear Import Of Rev Protein
Formation Of A Pool Of Free 40S Subunits
Influenza Infection

Figure S1

Fig. S1

a-f: Pathway enrichment analysis using enrichR of protein coding genes that have a significant, negative 3D-2D beta-score in the screens.

Table S1

miRNA	MCF7_2D_beta	MCF7_2D_FDR	MCF7_3D_beta	MCF7_3D_FDR	MCF7_3D-2D	HCC1395_2D_beta	HCC1395_2D_FDR	HCC1395_3D_beta	HCC1395_3D_FDR	HCC1395_3D-2D
miR-30e	-0.92987	0.17358	-1.3309	0.0076923	-0.40103	0.42179	0.99501	1.1248	0.66158	0.70301
miR-369	-0.30843	0.35409	-1.1016	0.040323	-0.79317	0.26982	0.88815	1.1315	0.65569	0.86168
miR-483	-0.79808	0.22114	-1.0852	0.042394	-0.28712	-2.0115	0	-0.6889	0.17295	1.3226
miR-4787	-0.32046	0.34572	-1.0758	0.043689	-0.75534	-0.72768	0.12372	-0.89082	0.098223	-0.16314
miR-130a	-0.28118	0.37672	-1.0468	0.050549	-0.76562	-0.03444	0.6022	-0.84546	0.11349	-0.81102
miR-1250	-0.34332	0.32812	-0.99826	0.060773	-0.65494	1.2197	0.30734	-0.14209	0.50444	-1.36179
miR-323a	-0.19842	0.46837	-0.99463	0.060773	-0.79621	0.11241	0.73959	0.14068	0.71507	0.02827
miR-6501	-0.77825	0.22627	0.045325	0.72525	0.82358	-0.65013	0.15946	-1.4833	0	-0.83317
miR-127	0.19614	0.81699	-0.021871	0.63031	-0.21801	0.34719	0.94803	-1.3694	0.0053191	-1.71659
miR-1468	-0.63907	0.27674	-0.97043	0.064286	-0.33136	-0.90634	0.077869	-1.3194	0.0053191	-0.41306
miR-485	-0.46378	0.29623	0.14241	0.85476	0.60619	-1.6963	0	-1.3064	0.012987	0.3899
miR-627	-0.61583	0.27822	-0.31148	0.29886	0.30435	0.19182	0.81707	-1.1517	0.040241	-1.34352
miR-301a	-0.69098	0.27822	-1.8878	0	-1.19682	0.54657	0.93453	-1.2724	0.06156	-1.81897
miR-4446	-0.10495	0.62028	-0.73486	0.11169	-0.62991	-0.28503	0.37572	-1.2378	0.029178	-0.95277

Table S1

Ranked negative ß-scores and FDR significance of miRNAs in 2D and 3D culture in MCF-7 and HCC1395 cells.

Breast Cancers METABRIC N=1262

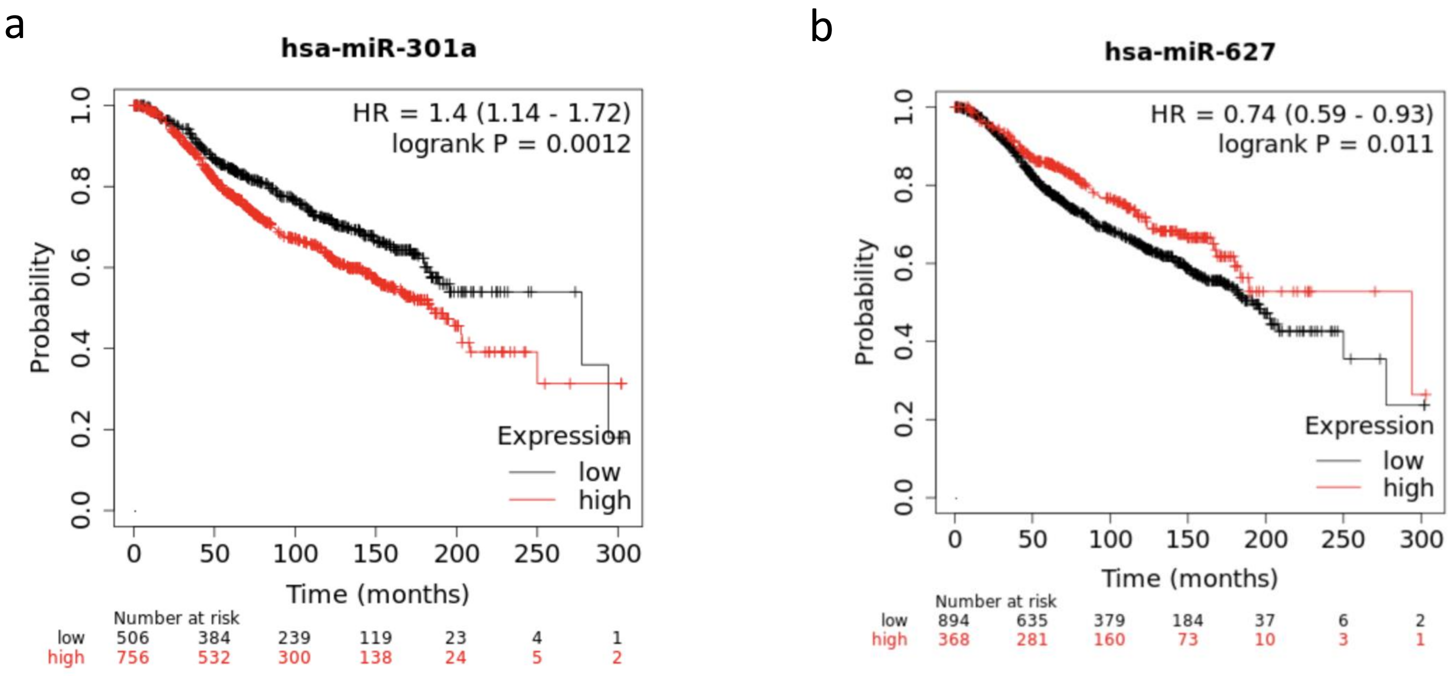

Fig. S2

a-b: Breast cancer METABRIC database Kaplan-Meier survival plots for miR-301a and miR-627.

Figure S3

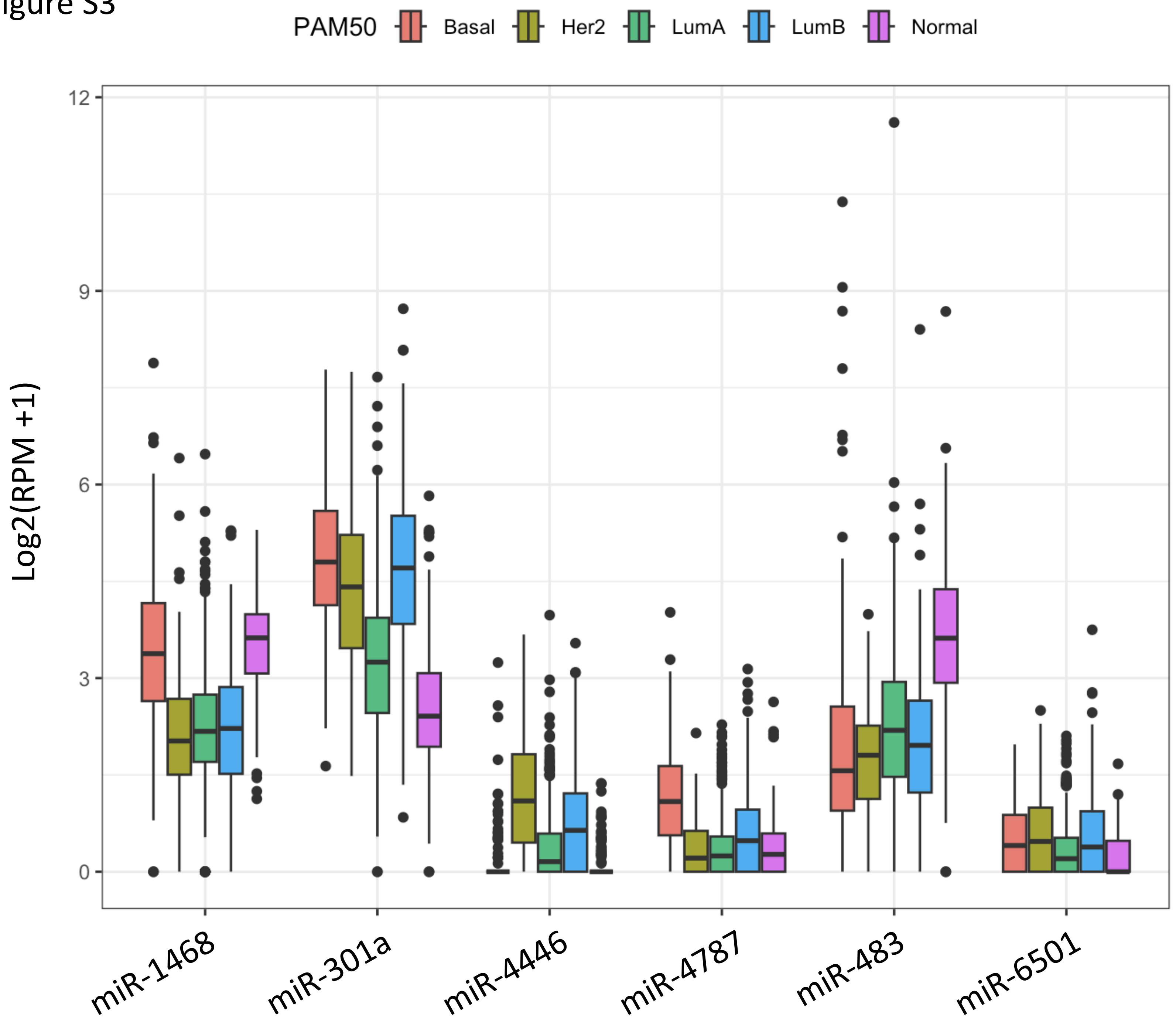

Fig. S3

Differential expression of miRNAs of interest in different BC molecular subtypes.

Figure S4

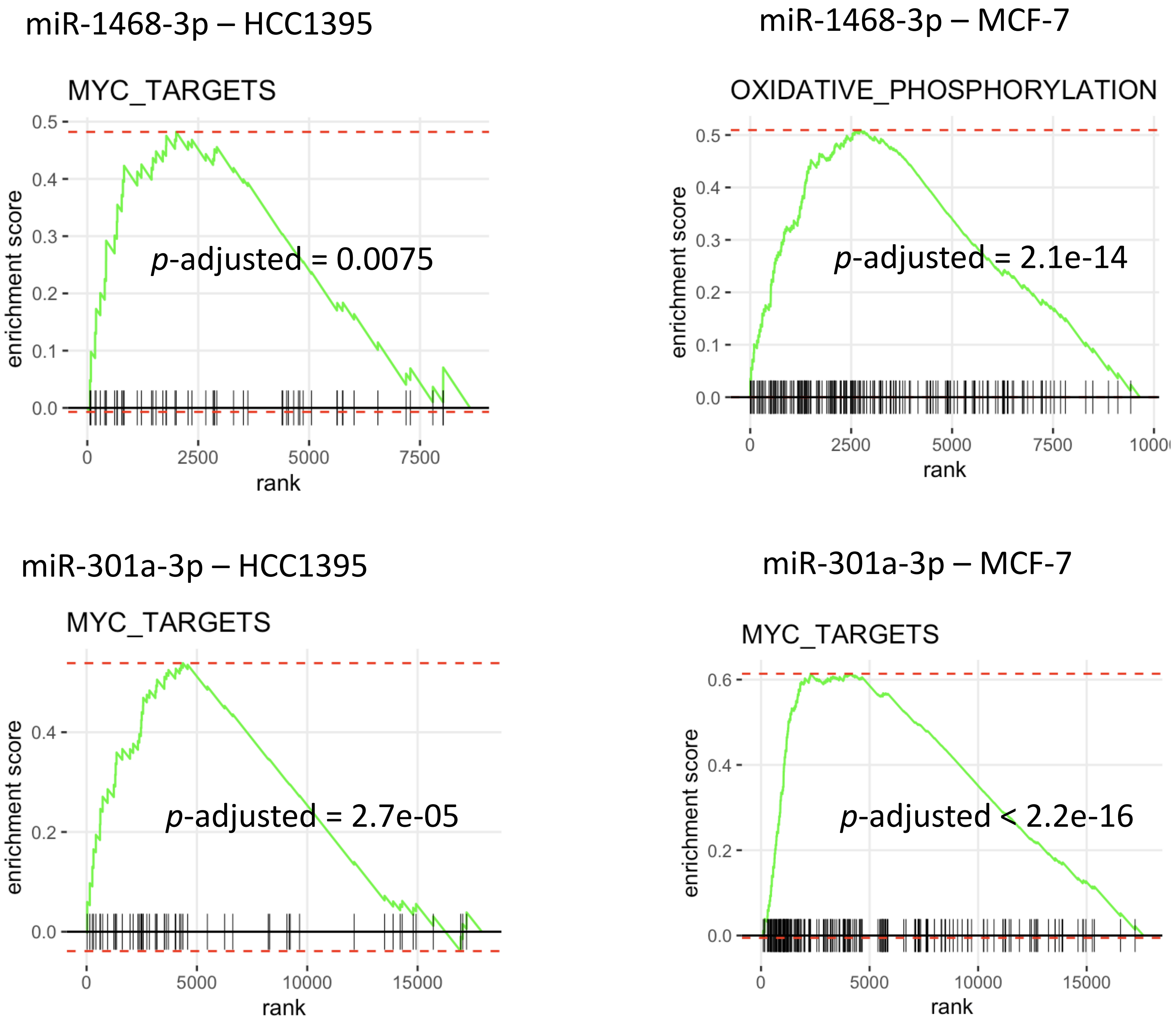
miR-1468-3p – HCC1395
MYC_TARGETS
enrichment score
rank
p-adjusted = 0.0075
miR-1468-3p – MCF-7
OXIDATIVE_PHOSPHORYLATION
enrichment score
rank
p-adjusted = 2.1e-14
miR-301a-3p – HCC1395
MYC_TARGETS
enrichment score
rank
p-adjusted = 2.7e-05
miR-301a-3p – MCF-7
MYC_TARGETS
enrichment score
rank
p-adjusted < 2.2e-16

Figure S4

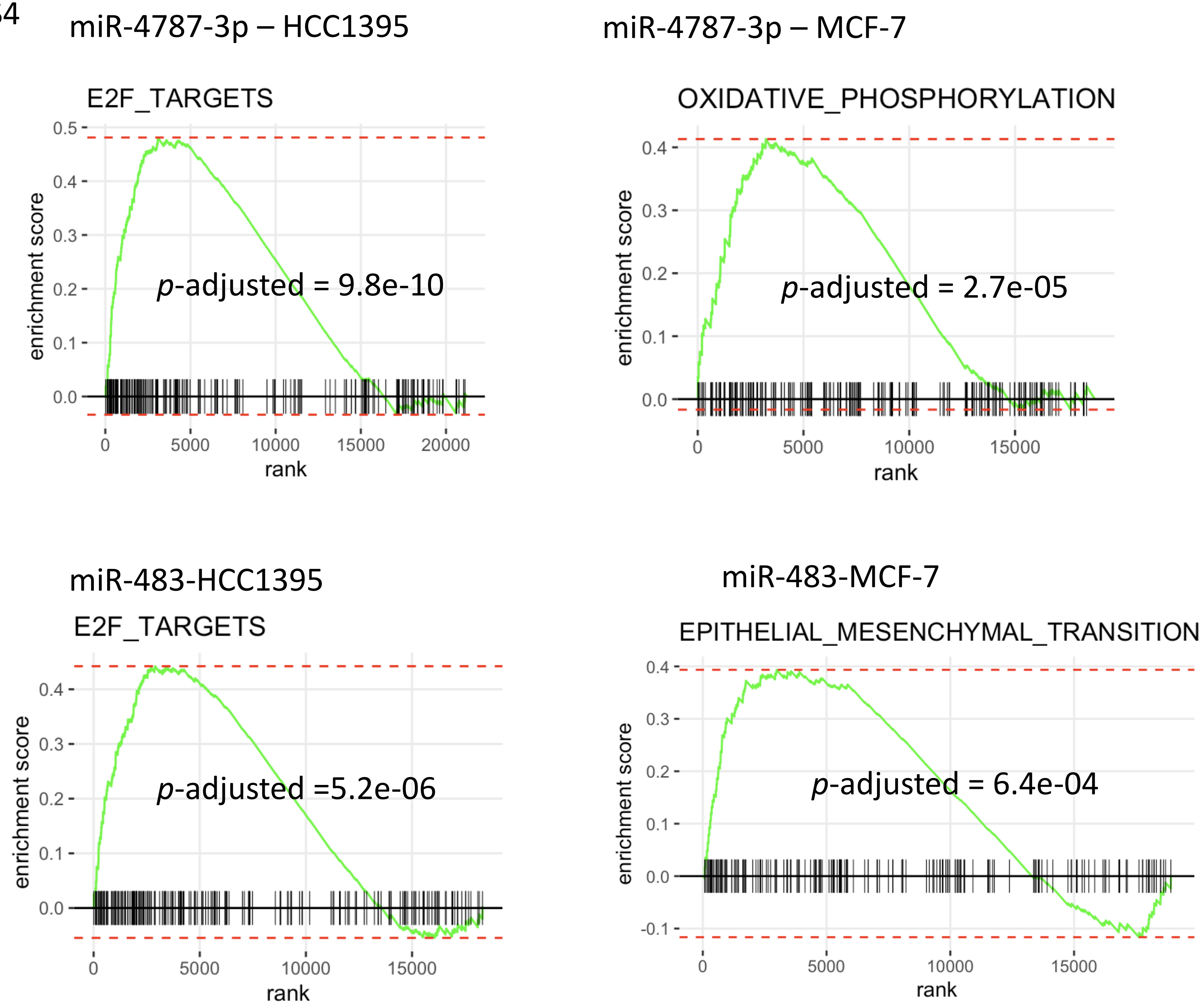
miR-4787-3p – HCC1395
E2F_TARGETS
p-adjusted = 9.8e-10
enrichment score
rank
miR-4787-3p – MCF-7
OXIDATIVE_PHOSPHORYLATION
p-adjusted = 2.7e-05
enrichment score
rank
miR-483-HCC1395
E2F_TARGETS
p-adjusted =5.2e-06
enrichment score
rank
miR-483-MCF-7
EPITHELIAL_MESENCHYMAL_TRANSITION
p-adjusted = 6.4e-04
enrichment score
rank

Figure S4

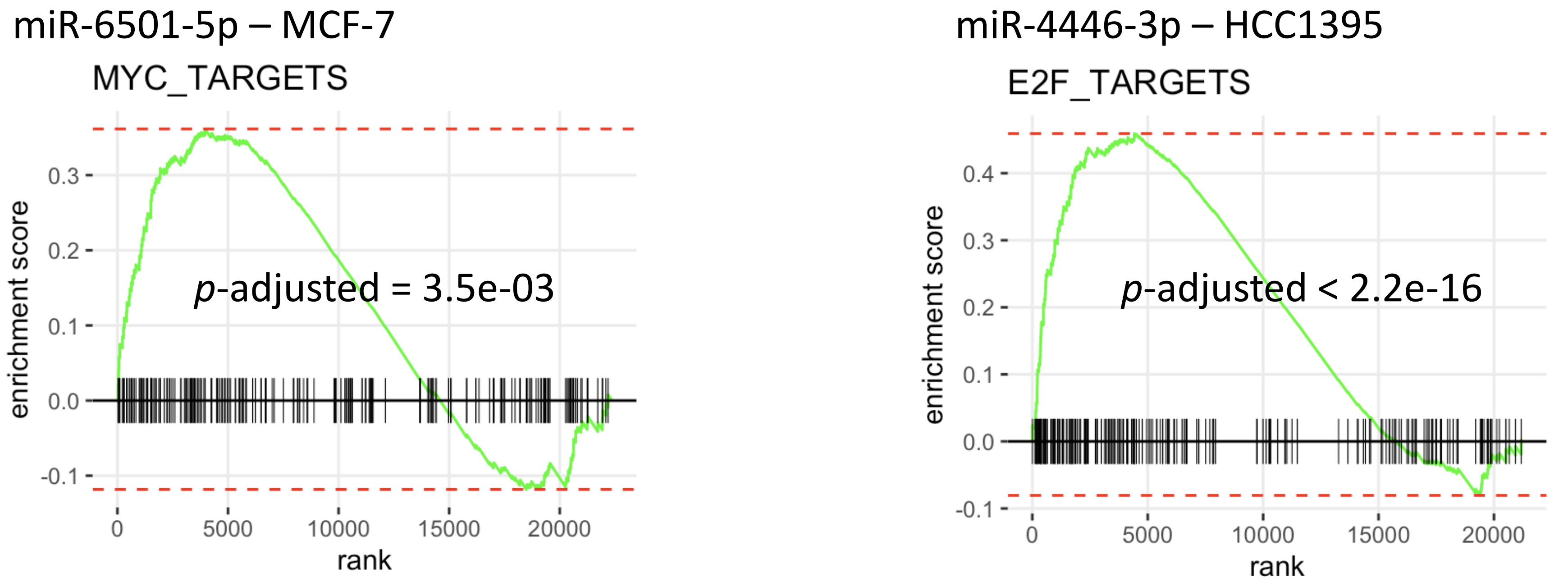

Fig S4

Gene set enrichment analysis (GSEA) after silencing of selected miRNAs following RNA-Seq in MCF7 or HCC1395 cell lines.

Figure S5

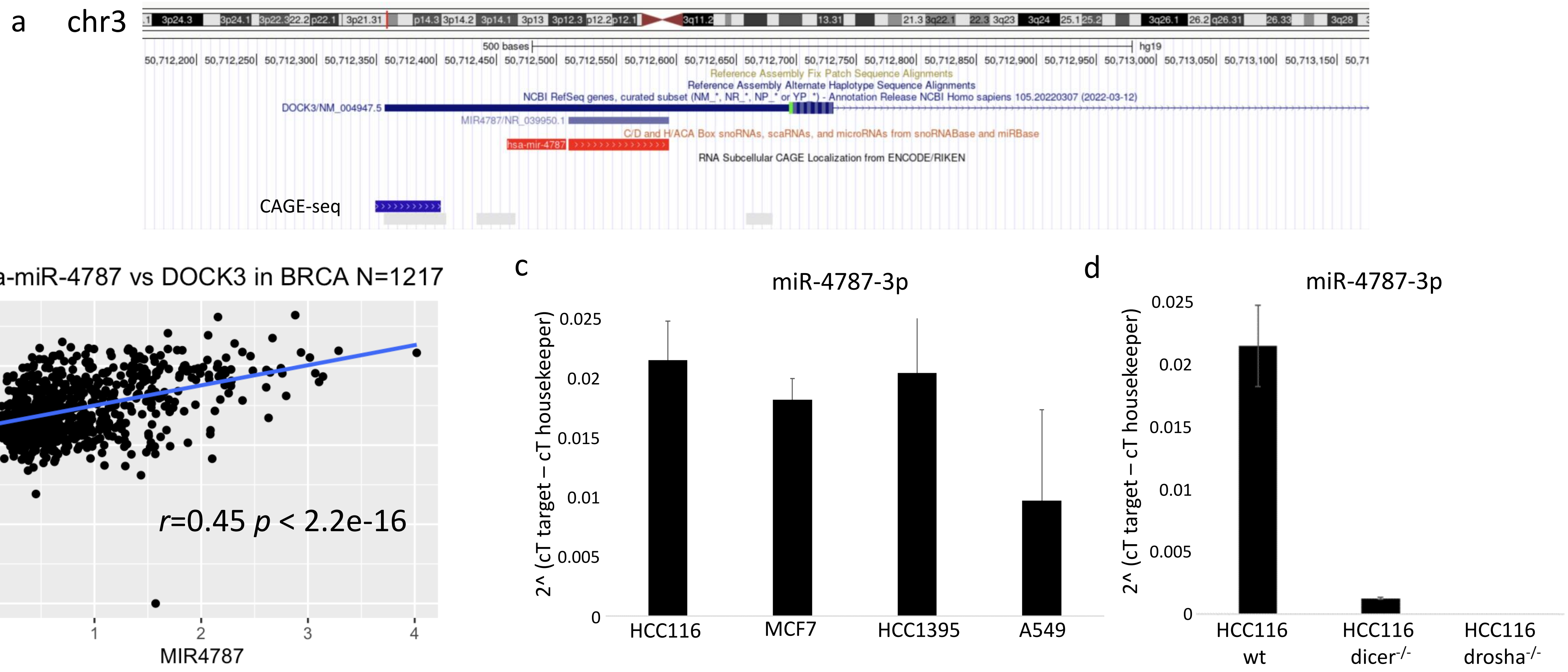

Fig. S5

a: miR-4787-3p genomic location maps closely and downstream of the transcriptional start site (TSS) of the protein-coding gene DOCK3.

b: Expression of miR-4787-3p and DOCK3 shows a positive correlation in BRCA specimens from the TCGA.

c: miR-4787-3p expression measured by qPCR in MCF-7, HCC3519 BC, HCT116 and A549 cancer cell lines. Graphs represent the average of 3 independent biological replicates.

d: miR-4787-3p expression measured in HCT116 cells that have been knockout (KO) for DROSHA or DICER1 through CRISPR/CAS9 editing. Graphs represent the average of 3 independent biological replicates.

Figure S6

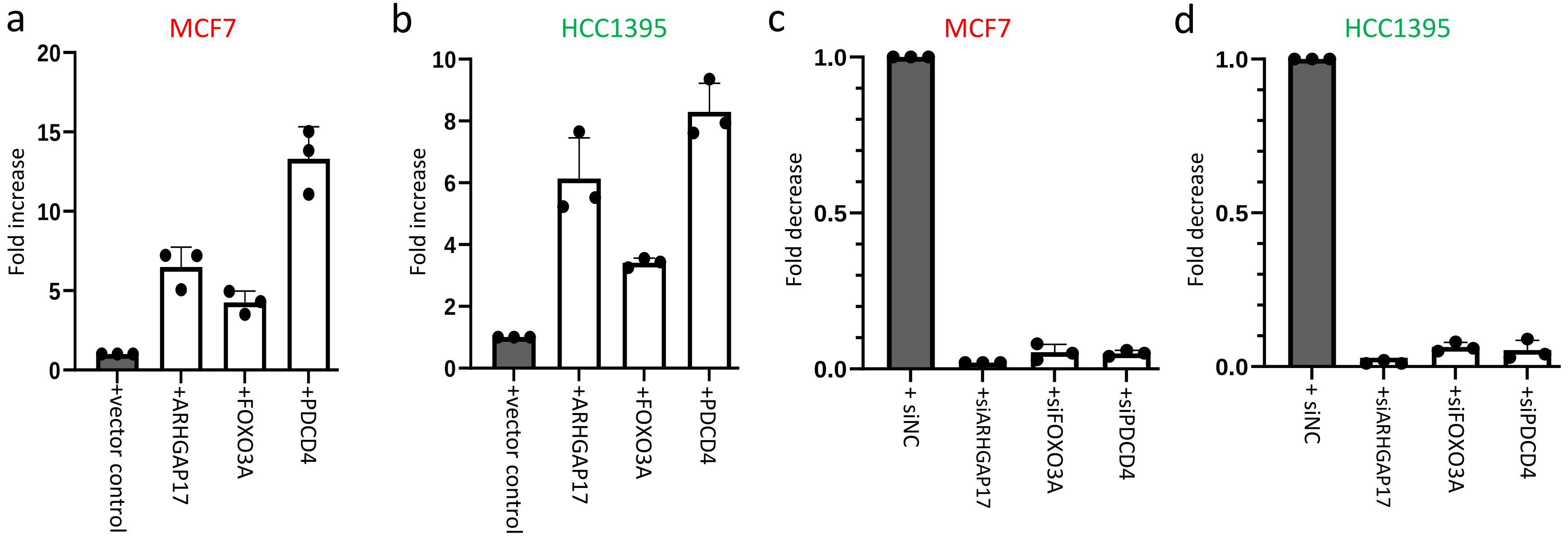

Fig. S6

a-b: qPCR analysis of overexpression of miR-4787-3p targets in MCF7 (a) and HCC1395 (b) cells normalised to vector control. For each condition 3 independent biological replicates were performed.

c-d : qPCR analysis of the inhibition of miR-4787-3p targets by siRNA compared to a negative control (siNC) in MCF7 (c) and HCC1395 (d) cells. For each condition 3 independent biological replicates were performed.
