# Supplementary material for: CRISPR screens in 3D tumourspheres identified miR-4787-3p as a transcriptional start site miRNA essential for breast tumour-initiating cell growth
Source: Commun Biol. 2024 Jul 13;7:859. doi: 10.1038/s42003-024-06555-1 (PMC11246431; doi:10.1038/s42003-024-06555-1)
Supplement: Supplementary file 3 — Description of Additional Supplementary Files [file 42003_2024_6555_MOESM3_ESM.pdf]

Description of Additional Supplementary Files

File name: Supplementary Data 1

Description: Model-Based Analysis of Genome Wide CRISPR-CAS9 Knockout (MAGeCK) analysis data set for CRISPR screen NGS data.

File name: Supplementary Data 2

Description: DESeq2 differential gene expression analysis data set.

File name: Supplementary Data 3

Description: Numerical data behind the graphs in the paper
