# Supplementary material for: CRISPR screens in 3D tumourspheres identified miR-4787-3p as a transcriptional start site miRNA essential for breast tumour-initiating cell growth
Source: Commun Biol. 2024 Jul 13;7:859. doi: 10.1038/s42003-024-06555-1 (PMC11246431; doi:10.1038/s42003-024-06555-1)
Supplement: Supplementary file 7 — Reporting Summary [file 42003_2024_6555_MOESM7_ESM.pdf]

Corresponding author(s): Leandro Castellano

Last updated by author(s): Jul 1, 2024

Reporting Summary

Nature Portfolio wishes to improve the reproducibility of the work that we publish. This form provides structure for consistency and transparency in reporting. For further information on Nature Portfolio policies, see our Editorial Policies and the Editorial Policy Checklist.

Statistics

For all statistical analyses, confirm that the following items are present in the figure legend, table legend, main text, or Methods section.

n/a	Confirmed	
☐	☒	The exact sample size (*n*) for each experimental group/condition, given as a discrete number and unit of measurement
☒	☐	A statement on whether measurements were taken from distinct samples or whether the same sample was measured repeatedly
☐	☒	The statistical test(s) used AND whether they are one- or two-sided<br>*Only common tests should be described solely by name; describe more complex techniques in the Methods section.*
☒	☐	A description of all covariates tested
☐	☒	A description of any assumptions or corrections, such as tests of normality and adjustment for multiple comparisons
☐	☒	A full description of the statistical parameters including central tendency (e.g. means) or other basic estimates (e.g. regression coefficient) AND variation (e.g. standard deviation) or associated estimates of uncertainty (e.g. confidence intervals)
☐	☒	For null hypothesis testing, the test statistic (e.g. *F*, *t*, *r*) with confidence intervals, effect sizes, degrees of freedom and *P* value noted<br>*Give P values as exact values whenever suitable.*
☒	☐	For Bayesian analysis, information on the choice of priors and Markov chain Monte Carlo settings
☒	☐	For hierarchical and complex designs, identification of the appropriate level for tests and full reporting of outcomes
☒	☐	Estimates of effect sizes (e.g. Cohen's *d*, Pearson's *r*), indicating how they were calculated

Our web collection on statistics for biologists contains articles on many of the points above.

Software and code

Policy information about availability of computer code

Data collection	No software was used
Data analysis	QuantSeq 3' mRNA-Seq Integrated Data Analysis Pipeline on Bluebee® (Lexogen) : www.lexogen.bluebee.com<br>TargetScan v8.0<br>Xena browser (https://xenabrowser.net).<br>R version 4.1.0 using the ggplot2 package version 3.3.6<br>pheatmap package version 1.0.12.<br>KMplot (https://kmplot.com/analysis/).<br>Enrichr (https://maayanlab.cloud/Enrichr/)<br>Gene Set Enrichment Analysis (GSEA) was run through the fgsea package version 1.18.0 in R.

For manuscripts utilizing custom algorithms or software that are central to the research but not yet described in published literature, software must be made available to editors and reviewers. We strongly encourage code deposition in a community repository (e.g. GitHub). See the Nature Portfolio guidelines for submitting code & software for further information.

Data

Policy information about availability of data

All manuscripts must include a data availability statement. This statement should provide the following information, where applicable:

- Accession codes, unique identifiers, or web links for publicly available datasets
- A description of any restrictions on data availability
- For clinical datasets or third party data, please ensure that the statement adheres to our policy

The RNA-seq and Gecko data were deposited in the NCBI Gene Expression Omnibus and are accessible through GEO SuperSeries accession number GSE243884 (https://www.ncbi.nlm.nih.gov/geo/query/acc.cgi?acc=GSE243884).
miRNA expression from the TCGA
Stem loop miRNA expression counts transformed to log2(RPM + 1) values were obtained from the GDC TCGA BC datasets from the University of California Santa Cruz (UCSC) Xena database (http://xena.ucsc.edu).

Human research participants

Policy information about studies involving human research participants and Sex and Gender in Research.

Reporting on sex and gender	n/a
Population characteristics	n/a
Recruitment	n/a
Ethics oversight	n/a

Note that full information on the approval of the study protocol must also be provided in the manuscript.

Field-specific reporting

Please select the one below that is the best fit for your research. If you are not sure, read the appropriate sections before making your selection.

☒ Life sciences ☐ Behavioural & social sciences ☐ Ecological, evolutionary & environmental sciences

For a reference copy of the document with all sections, see nature.com/documents/nr-reporting-summary-flat.pdf

Life sciences study design

All studies must disclose on these points even when the disclosure is negative.

Sample size	No statistical methods were used to predetermine sample sizes; however, the sample sizes were comparable to those in prior studies. Statistical methods were utilized to calculate p values. Upon applying the cutoffs for p values and fold changes, we identified changes of statistical significance as anticipated.
Data exclusions	No data excluded
Replication	All attempts at replication were successful. At least 3 biological replicates were performed
Randomization	We did not carry out randomization as this is either irrelevant or not applicable to this study.
Blinding	Blinding was not possible for cell culture experiments because a single experimenter carried out the experiments. Samples sent for NGS for Gecko library DNA sequencing or RNA-seq were blinded by experimenter and decoded after analysis.

Reporting for specific materials, systems and methods

We require information from authors about some types of materials, experimental systems and methods used in many studies. Here, indicate whether each material, system or method listed is relevant to your study. If you are not sure if a list item applies to your research, read the appropriate section before selecting a response.

Materials & experimental systems

n/a	Involved in the study
☒	☐ Antibodies
☐	☒ Eukaryotic cell lines
☒	☐ Palaeontology and archaeology
☒	☐ Animals and other organisms
☒	☐ Clinical data
☒	☐ Dual use research of concern

Methods

n/a	Involved in the study
☒	☐ ChIP-seq
☒	☐ Flow cytometry
☒	☐ MRI-based neuroimaging

Eukaryotic cell lines

Policy information about cell lines and Sex and Gender in Research

Cell line source(s)	Authenticated cell lines MCF7 and HCC1395 were obtained from the Genome Damage and Stability Centre (Sussex) Cell Bank.
Authentication	Cell bank lines were authenticated by ECACC using short tandem repeat (STR) profiling.
Mycoplasma contamination	All cell lines used tested negative for mycoplasma (MycoAlert, Lonza)
Commonly misidentified lines (See ICLAC register)	No commonly misidentified lines were used in this study
